# *Swarna Bhasma* reduces the blood concentration of tumor-specific signatures and protects from hepatocellular damages in Ehrlich ascites mice model

**DOI:** 10.1016/j.jaim.2025.101152

**Published:** 2025-07-24

**Authors:** Namrata Joshi, Pankaj Kumar, Shiwakshi Sharma, Remya Jayakumar, Anand Mishra, V. Harsha, Manoj Kumar Dash

**Affiliations:** a Department of Rasa Shastra and Bhaishajya Kalpana, Faculty of Ayurveda, Institute of Medical Sciences, Banaras Hindu University, Varanasi, 221005, India; b Department of Anatomy, Institute of Medical Sciences, Banaras Hindu University, Varanasi, 221005, India; c Department of Rasa Shastra and Bhaishajya Kalpana, Shri Narayan Prasad Awasthi Government Ayurved College, Raipur, Chhattisgarh, 492010, India

## Abstract

****Background**:**

The paradigm shift with alarmingly high rate of global cancer incidences encourages the application of incinerated gold Nano powder, Swarna Bhasma (SB) due to its exceptional potency, affordability, and minimal toxic effects. Previous experimental investigations were unable to provide a biochemical understanding of the anti-carcinogenic properties of SB.

****Objective**:**

To evaluate the tumour related markers in blood and possible alteration in hepatic parameters due to SB.

****Methods**:**

EAC (Ehrlich’s Ascites Carcinoma) induced tumour was generated in the female Swiss albino mice divided into 6 groups, namely, Vehicle Control (VC), Disease Control (DC), Standard Control (SC), and Treatment Groups with escalating doses (1.95, 3.9, and 7.8 mg/kg body weight) of SB. Blood serum quantified was measured for the levels of CEA (Carcinoembryonic antigen), TNF-α (Tumour Necrosis Factoralpha), IL-6 (Interleukin-6), ALT (Alanine transaminase), and AST (Aspartate aminotransferase). Changes in daily food consumption, body weight, and tumour volume (with Vernier caliper) were coherently studied and analysed. The data was analysed using One-Way ANOVA and Tukey's Honest Significance Test.

****Result**:**

SB demonstrated effective reduction of CEA levels at higher doses, and TNF-α levels at medium doses. Both moderate and high doses exhibited a noteworthy, dosedependent decrease in IL-6 levels. Furthermore, SB led to a dose-dependent reduction in the AST/ALT ratio. A significant reduction in tumour volume were reported in both the moderate and high doses of SB along with marked improvement in anorexia. The higher doses of SB exhibited the serum validated results in the hepatic, renal and the splenic tissues.

****Conclusion**:**

The anti-carcinogenic activity of SB appeared to be dose-dependent. The finding also underscored the hepato-protective capability of SB in lower dose by alleviating cancer-related liver damage

## Highlights

• *Swarna bhasma (SB)* exhibited an average size of 7.67 nm with spherical and oval-shaped particles as per analytical outcomes.
• SB in the EAC mice model reduced the tumor-specific markers (CEA, TNF-α, IL-6).
• The escalating doses of SB reflected different variations among the hepatic blood parameters (ALT, AST, and AST/ALT ratio).

## 1 Introduction

In the ancient Indian sites of the Indus Valley Civilization and the Vedic ages, there is a notable absence of paleo-oncology reports documenting cases of cancer [1]. Likewise, the term "cancer" itself remains absent from the texts of that era. However, references to ailments resembling cancer, such as *Arbuda*, *Granthi*, and *Gulma*, can be found in the revered Ayurveda classics [2]. These texts primarily focus on more prevalent ailments, hinting at the relatively rare occurrence of cancer during those bygone times.

In the contemporary landscape, cancer has emerged as a formidable non-communicable disease, intricately intertwined with lifestyle factors, presenting a profound global health challenge [3]. Astonishingly, the probability of cancer, especially the occurrence of breast cancer in India is a subject of prime concern among various age groups [4]. On a global scale, the landscape of cancer incidence has witnessed a paradigm shift, with female breast cancer surmounting lung cancer as the most prevalent malignancy [5]. The genesis of cancer lies in aberrant cellular metabolism driven by elusive causes. In the current clinical landscape, one of the most efficacious strategies involves targeting the pivotal functional pathways responsible for the onset of cancer. Several landmark efforts are made through synthesis of diverse forms of NPs by using cutting edge technologies to target the multifaceted challenges posed by this disorder [6,7]. Presently, computational approach, interference of cellular signaling pathways in cancer by natural products, pharmacogenetics and molecular signal transduction are some of the strategies that are being pursued in the field of cancer research [8]. However, the traditional approach takes a distinctly different route. Within Ayurveda, an ancient medicinal tradition, both herbal and herb-mineral methodologies are commonly embraced to address cancer like symptoms. In the realm of Ayurveda, an intriguing medicinal class exists alongside botanical remedies [9,10]. This category delves into the realm of metals and minerals, providing a unique approach to healing. Within this domain, the remarkable ayurvedic nanomedicines take center stage, harnessing the power of metal Nano-powder, colloquially known as *Bhasma*. These *Bhasma* formulations are revered for their natural process of origin, exceptional potency, coupled with minimal adverse effects when administered according to prescribed classical guidelines [[11], [12], [13]].

*Swarna bhasma* (SB), a herbo-mineral formulation based on gold, holds significant pharmacotherapeutic value. Ancient Ayurvedic texts describe various properties of gold, and SB is particularly employed for chronic diseases, rejuvenation, mental disorders, and many other intricate health disorders including the Diabetes mellitus syndrome where the *Vasantakusumakara rasa* like formulations are seen prescribed [14]. SB nanoparticles (NPs) exhibit unique characteristics, including *rasayana* (immune regulation and anti-aging), *yogavahi* (targeted delivery of drug), *alpamatra* (recommended in small doses), *rasibhava* (rapid and easy absorption, adaptability, assimilation, and non-toxicity), *shigravyapi* (quick distribution and rapid action), and *agnideepana* (enhancement of cellular metabolism and catalyst-like activity) [15]. Contemporary science using the Green Nano synthesize technology has been able to produce economically sustainable NPs. Moreover, the synthesis of AuNPs via green technology have vast surface area with the phytochemical encapsulation resembling the traditional SB preparation. The application of Nano-Ayurvedic medicine on the MDA-MB-231 cell line, SCID female mice and further the pilot study revealed high selectivity on tumour cells, capacity to reduce the tumour volume and in the pilot study the combination treatment exhibited better results [16]. Whereas, in another experiment, the mangiferin encapsulated AuNPs on PC-3 cells reduced the IL-6 and IL-10 (pro-tumor) and increased the TNF-α and IL-12 expression in the treated groups [17].

The *Bhasma* Nano particles (BNPs) possesses the versatility of no necessity for further surface-related alterations which is a common practice seen in traditional synthetic drugs aimed at amplifying therapeutic effects. This is a direct consequence of an intermediate pharmaceutical process known as *Bhavana*. The aftermath of the procedures is a spontaneous metamorphosis with an envelope of the nanocrystalline metallic core and a resplendent corona, embellished with a myriad of trace elements. Similarly, being synthesized through specific natural 3000 years old traditional technique with integration of herbal bioactive molecules by bhavana process, SB NPs are found biocompatible, non-toxic, and non-antigenic, making them suitable for selective, targeted, or controlled drug administration in cytotoxic contexts Extensive research has validated the classical claims, demonstrating the diverse effects of SB NPs, including anti-inflammatory and immunomodulatory effects, antioxidant activity, clearing excess free radical, analgesic properties, and anti-cataleptic, anti-anxiety, and anti-depressant activities [18]. Despite the well-documented effects, the biochemical interactions between SB NPs and cellular components remain incompletely understood and present a fertile area for active research. Based on the discussed pharmacological perspectives, the current experimental study investigates the potential anti-carcinogenic properties of SB using selected blood-related biochemical parameters.

## 2 Material and method

### 2.1 Raw material

Gold biscuits of 99.9 % purity (MMTC-PAMP brand) were procured for the research. All the raw drugs of herbal origin were obtained from a local market and authenticated at the Department of Botany, Institute of Science, Banaras Hindu University, Varanasi, following the *shodhan* process defined in classical texts. The raw drugs from animal origin were procured from a dairy farm at the Institute of Agricultural Sciences, BHU, Varanasi.

### 2.2 Preparation of Swarna bhasma

**Fig. 1:**
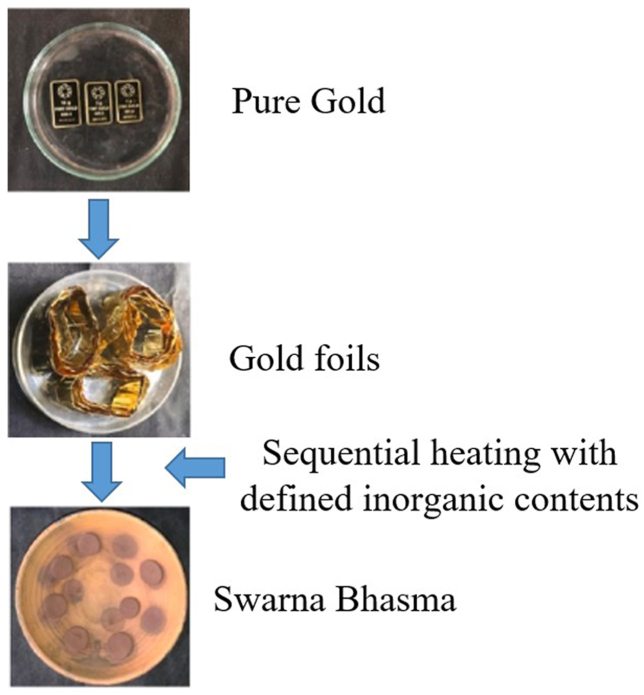
Preparation of Swarna bhasma (SB). Pure gold was transformed into foil to expose its surface. The conventional purification method was applied, followed by blending it with classically defined inorganic components (in this case, purified mercury and sulfur). Subsequently, this mixture underwent a series of distinct heat treatments in a specific manner. After numerous tries, the ultimate product, Swarna bhasma, possessing the desired characteristics, was achieved. (For interpretation of the references to colour in this figure legend, the reader is referred to the Web version of this article.)

The gold biscuits were meticulously converted into delicate foils, possessing a thinness that allowed them to be easily punctured by a needle. These foils then underwent a classical purification procedure, adhering to the guidelines outlined in *Rasa Tarangini* [19]. After that the processed gold was carefully passed through sequential alchemical procedures to prepare *Swarna bhasma* (SB) (Fig. 1).

### 2.3 Drug characterization

**Fig. 2:**
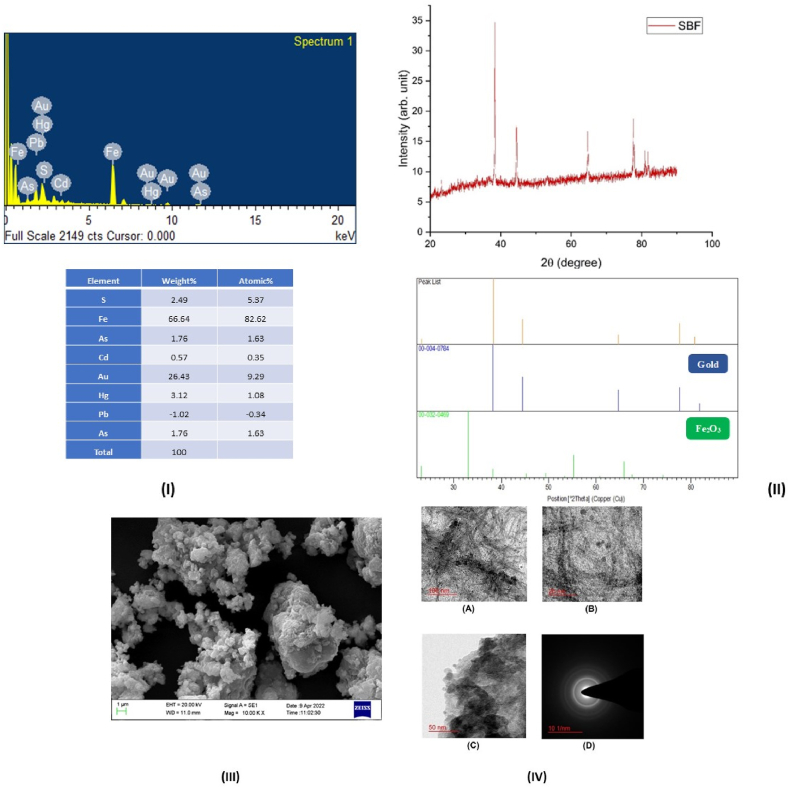
EDAX, XRD and TEM Image of Swarna Bhasma. (I) EDAX mapping with elemental abundance of final product SBF (Swarna Bhasma), (II) graph of sample SBF and peaks of corresponding elements/compounds, (III) SEM images of SBF, at 10k magnification, (IV) TEM images of sample SBF. The magnification of (A), (B), and (C) are 50k, 200k, and 100k respectively. (D) Diffraction pattern in dark field shown in having camera length 120 mm.

The characterization of SB involved the utilization of established techniques, including X-ray Diffraction (XRD), Scanning Electron Microscopy (SEM), Energy Dispersive X-Ray Spectroscopy Analysis (EDXA), and Transmission Electron Microscopy (TEM), to investigate its physicochemical properties. XRD exhibited distinct peaks corresponding to gold and various other elements/compounds, highlighting their presence within SB. The application of the Scherrer equation allowed for the estimation of particle size, revealing an average size of approximately 15.24 nm for the final product. The observed peaks exhibited exceptional sharpness, indicating a crystalline nature. The elemental composition of SB, expressed as weight percentage, was found to be 26.43 % gold, 45.38 % sulfur, 66.64 % iron, and 3.12 % mercury. The TEM analysis further elucidated the structure of SB, uncovering the existence of finely dispersed spherical and oval-shaped particles with an average size of 7.67 nm (Fig. 2) [20].

### 2.4 Experimental model

Female Swiss albino mice, aged between 6 and 10 weeks and weighing approximately 25–30 g, were specifically chosen as the subjects for this study. A total of 36 mice were procured from the Central Animal House at the Institute of Medical Sciences, Banaras Hindu University. The commencement of the study was contingent upon obtaining clearance from the Institutional Animal Ethical Committee, with a designated certification number of Dean/2021/IAEC/2545. Additionally, the study adhered to the guidelines set forth by the CCSEA Registry, under the registration number 542/GO/ReBi//S/02/CCSEA, dated May 26, 2017.

The mice were housed in a controlled environment, maintaining a temperature of 25 °C (±3 °C) and an artificial lighting setup that followed a 12-h cycle of light and darkness. Efforts were made to ensure the relative humidity ranged between 40 % and 70 %. Each Polypropylene cage accommodated 6 mice, and their water supply was provided through tap water. The mice were fed VRL brand pellets as their primary food source, with access to drinking water *ad libitum*.

### 2.5 Experimental design

The original Ehrlich Ascites Carcinoma (EAC) cell line, stored in a cryogenic state in liquid nitrogen, was used to induce tumors in mice groups by following a well-established protocol as outlined in previously conducted studies [20,21]. The EAC cells from the Department of Biochemistry were revived from vials by diluting with 10 % Phosphate Buffer Solution (PBS) and the temperature of the vial was set to room temperature. This EAC (1 × 10^6^ cells) solution was inoculated initially in six mice and when the ascites symptoms developed (7–10 days), the fluid from them are injected to the flanks of the mice. Once the tumor became palpable, all the mice were randomly allocated to one of six groups: Group I - Vehicle Control (VC), Group II - Disease Control (DC), Group III - Standard Control (SC), and Groups IV, V, and VI - Treatment groups with escalating doses. Honey was utilized as the vehicle, administered orally at a dose of 500 mg/kg. The disease control group did not receive any treatment. Doxorubicin 50 was used as a standard drug (Table 1). The dosage of the drug in mice was determined by extrapolating the therapeutic dose for humans using the conversion factor of 0.0026 [22]. For a 20g mouse, the calculated dose of SB was 3.9 mg/kg of body weight.

**Table 1:** Animal group design for the experimental study.

Group No.	Group Name
Group I	Vehicle control (EAC + honey 5 ml/Kg body wt.)
Group II	Disease control (EAC)
Group III	Standard control EAC + (Doxorubicin 50, 2 mg/Kg body wt.)
Group IV	Treatment group (SB 1.95 mg/kg in honey 5 ml/Kg body wt)
Group V	Treatment group (SB 3.9 mg/kg in honey 5 ml/Kg body wt)
Group VI	Treatment group (SB 7.8 mg/kg in honey 5 ml/Kg body wt)

### 2.6 Bio-physical and -chemical analysis

Blood serum samples were collected from each mouse in every group before they were sacrificed. These serum samples were then subjected to analysis using an automatic biochemistry analyzer to determine various essential blood parameters. Specifically, the focus of the study was on assessing tumor markers and associated chemical parameters. This involved conducting tests to measure the levels of carcinoembryonic antigen (CEA), tumor necrosis factor-alpha (TNF-α), interleukin-6 (IL-6), as well as alanine aminotransferase (ALT) and aspartate aminotransferase (AST) enzymes. These evaluations were performed to explore the effect of the experimental conditions on these specific biomarkers and biochemical indicators. Biophysical parameters, such as body weight, daily food consumption, and tumour volume (V = 0.5 × L × W^2^, where V is volume, L is length and W is width, with Vernier Caliper) were also analyzed in this experiment to check the coherent similarities with biochemical determinants.

### 2.7 Statistical analysis

To assess the potential effectiveness of the treatment against the disease, a One-Way ANOVA analysis was conducted (Table 2). This statistical test was utilized to determine if there were substantial variations between the groups being compared. If the ANOVA result yielded a significant finding, indicating that there were notable variations between the groups, further analysis using Tukey's Honest Significance Test was performed. p < 0.05 is marked as significant (∗p < 0.05; ∗∗p < 0.01; ∗∗∗p < 0.001; ∗∗∗∗p < 0.0001). Tukey's test is a post-hoc analysis that allows for the evaluation of the degree of significance between specific pairs of groups (Table 3).

**Table 2:** Comparative data of all groups- CEA, TNF-α, IL-6, AST/ALT (ANNOVA Test).

Parameters	Group 1 (VC)	Group 2 (DC)	Group 3 (SC)	Group 4 (TL)	Group 5 (TM)	Group 6 (TH)	P -value	Result
**CEA**	1.5175 ± 0.1905	2.1275 ± 0.1891	0.6300 ± 0.0642	2.5425 ± 0.4330	1.2450 ± 0.5871	0.5900 ± 0.0668	<0.01	∗∗
**TNF-α**	11.1750 ± 0.4476	18.2500 ± 1.8120	8.20000 ± 0.9255	14.1500 ± 2.8875	9.3000 ± 1.2293	10.9950 ± 1.4496	<0.01	∗∗
**IL-6**	28.7875 ± 0.8943	32.1200 ± 0.6198	11.6750 ± 0.6981	37.2250 ± 5.7488	12.8000 ± 0.7223	11.0875 ± 11.2746	<0.0001	∗∗∗∗
**AST/ALT**	3.2572 ± 0.5066	2.0257 ± 0.2332	2.8015 ± 0.6560	3.4605 ± 0.1560	7.0060 ± 3.1356	10.2525 ± 2.9903	<0.05	∗

**Table 3:** Comparative results after applying Tukey's Honest Significance Test between groups in relation to CEA, TNF--α, IL-6, and AST/ALT.

Parameters	Group 1vs Group 2	Group 1vs Group 3	Group 1vs Group 4	Group 1vs Group 5	Group 1vs Group 6	Group 2vs Group 3	Group 2vs Group 4	Group 2vs Group 5	Group 2vs Group 6	Group 3vs Group 4	Group 3vs Group 5	Group 3vs Group 6	Group 4vs Group 5	Group 4vs Group 6	Group 5vs Group 6
**CEA**	>0.05	>0.05	>0.05	>0.05	>0.05	<0.05	>0.05	>0.05	<0.05	<0.01	>0.05	>0.05	>0.05	<0.01	>0.05
**TNF-α**	>0.05	>0.05	>0.05	>0.05	>0.05	<001	>0.05	<0.05	>0.05	>0.05	>0.05	>0.05	>0.05	>0.05	>0.05
**IL-6**	>0.05	<0.01	>0.05	<001	<0.01	<0.001	>0.05	<0.001	<0.001	<0.0001	>0.05	>0.05	<0.0001	<0.0001	>0.05
**AST/ALT**	>0.05	>0.05	>0.05	>0.05	>0.05	>0.05	>0.05	>0.05	<0.05	>0.05	>0.05	>0.05	>0.05	>0.05	>0.05

## 3 Result and discussion

**Fig. 3:**
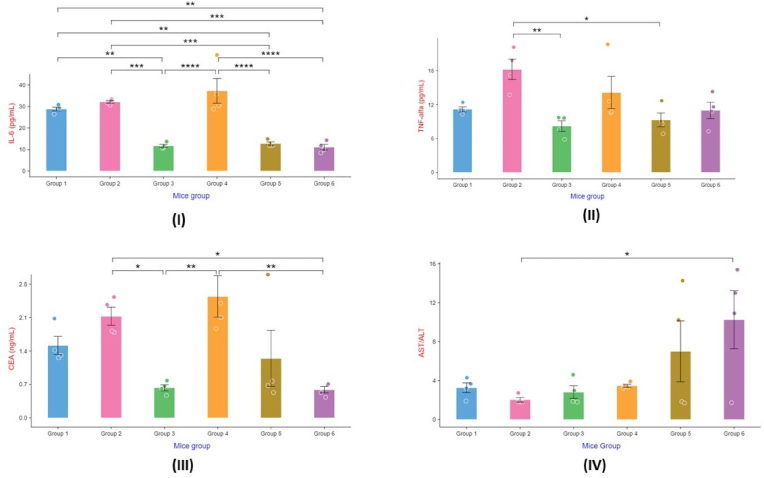
Effect of different treatment of Swarna Bhasma on the various biochemical parameters (I) IL-6, (II) TNF-α, (III) CEA, and (IV) AST/ALT.

**Fig. 4:**
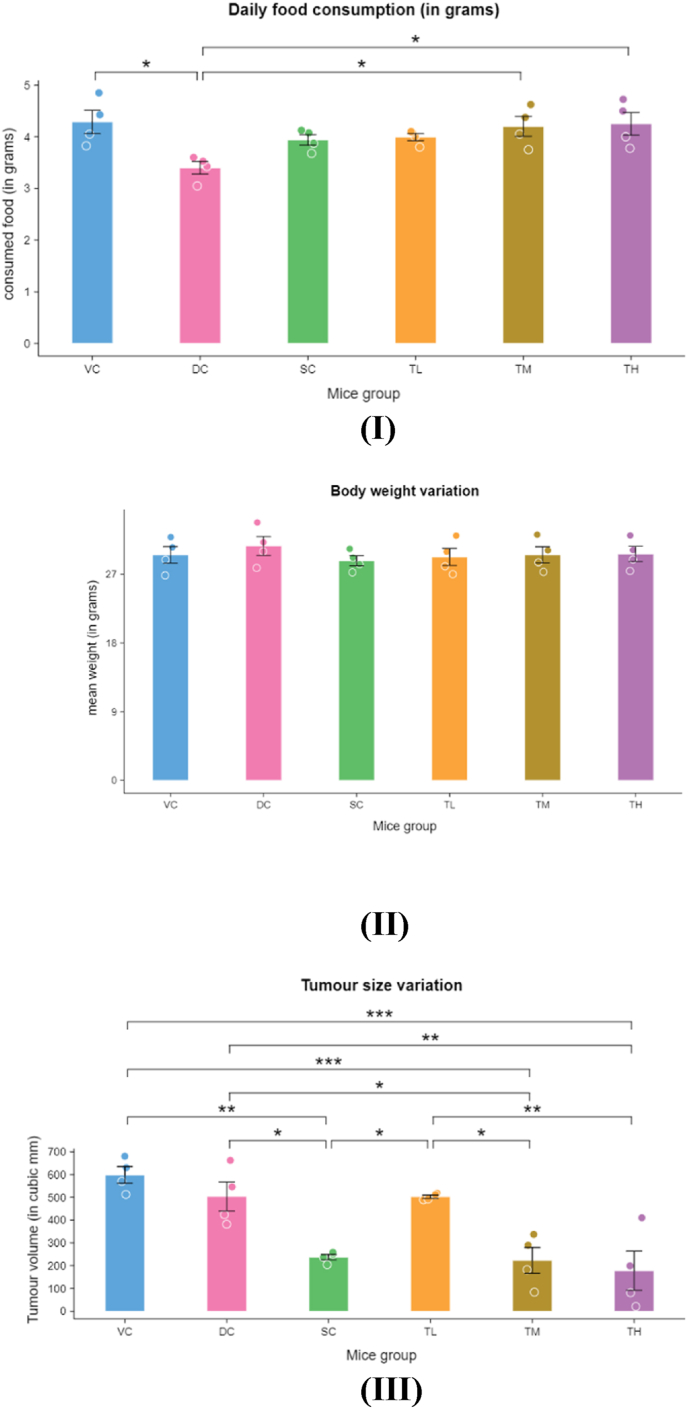
Variation in biophysical parameters induced by different treatment dose of *Swarna Bhasma* (I) Daily food consumption, (II) mean body weight, and (III) tumour size.

*Swarna Bhasma* (SB) has been treated as a universal drug because of being applied in several types of pathologies including malignant and non-malignant growth. Among these classical claims, the anti-tumour potential of SB was investigated in this *in vivo* study. The experiment revealed that SB at higher dose (7.8 mg/kg body weight) can be considered as a potent anti-carcinogenic drug as it helped in significant reduction of the haematological parameter such as Carcinoembryonic antigen (CEA), Tumor Necrosis Factor (TNF Alpha), and Interleukin-6 (IL-6), while SB at medium dose (3.9 mg/kg body weight) helped in reducing only IL-6 parameter suggesting its mild anti-carcinogenic potential. Low dose (1.95 mg/kg body weight) of SB showed no anti-carcinogenic effect but helps in promoting the hepatic functions against the disease (Fig. 3). Also, the mean daily food consumption of both the mice groups TM and TH had found significant high in comparison to the DC group. Reduction in tumour size (volume) was very significant in both the TM and TH groups in comparison to VC group, while it was significant when compared with DC group. (included in Table 4 in Supplementary file). There was no significant variation in mean weight of the mice groups from day 0 to day 21 between either of the treated or control groups (Fig. 4). The hepatic slides also exhibited this feature due to the sunrise appearance was seen in the TH group when the SC or the *Swarna Bhasma* (SB) treated other groups were lacking the same.

As gold nanoparticles (GNPs), the nanoparticles derived from SB, possess distinctive and intriguing physicochemical properties, including surface plasmon resonance (SPR) and the selective binding ability to amine and sulfhydryl groups [23]. These remarkable characteristics enable SB nanoparticles (NPs) to engage in a myriad of biochemical interactions and elicit multifaceted physiological responses [24]. Regarding the cellular internalization mechanism of GNPs, non-specific receptor-mediated endocytosis (RME) is believed to be the most plausible route [[25], [26], [27], [28]]. Remarkably, even without specific targeting ligands, the NPs passively accumulate within tumor sites, taking advantage of the leaky and immature vasculature characterized by significantly wider fenestrations (over 30 times larger than those of normal mature blood vessels) [29]. This phenomenon, known as the enhanced permeability and retention (EPR) effect, is coupled with prolonged circulation times facilitated by the hydrophobic nature of SB and the evasion of cellular homeostasis mechanisms for essential elements. Consequently, this synergistic interplay between the EPR effect and prolonged circulation times can substantially enhance the drug concentration within tumors by 10–100 times compared to the administration of free medicines [30]. In this context, the pharmacological effects of the drugs primarily target cell surface-based receptors, including notable examples such as carcinoembryonic antigen (CEA).

CEA, a ubiquitous cell surface glycoprotein, holds significant value as a prognostic and tumor biomarker. Its overexpression is observed in a wide array of cancer types, encompassing approximately 90 % of abdominal malignancies, 70 % of pulmonary carcinoma, and approximately 50 % of breast cancers. CEA plays a pivotal role in mediating homotypic intercellular adhesion, and its overproduction actively contributes to carcinogenesis by disrupting normal tissue architecture, resulting in inhibited differentiation and tissue dysplasia. In our study, we observed that higher doses of SB effectively reduced CEA levels, comparable to the standard drug (Fig. 2), thereby showcasing the anticancer properties of SB. Furthermore, CEA synergistically interacts with Myc and Bcl-2 in cellular transformation. The simultaneous expression of v-myc, bcl-2, and CEA in cells exhibited all the characteristic features associated with a fully transformed phenotype, including increased cellular division, higher saturation density, diminished apoptosis, absence of differentiation, and prolonged survival [31]. Within this triangular relationship, bcl-2 expression fully suppresses apoptosis, while v-myc, mimicking c-myc, induces the formation of tumor-initiating anchorage-independent colonies composed of undifferentiated cells, in conjunction with CEA [32]. Extensive research has demonstrated the significant influence of GNPs on the expression of c-myc, a gene that is dysregulated in up to 70 % of human cancers. GNPs were observed to accumulate within the mitochondria of tumor cells, leading to their destruction and dysfunction. This process induces excessive oxidative stress, resulting in the generation of excessive reactive oxygen species (ROS) and triggering mitochondria-mediated apoptosis in tumor cells. Moreover, GNPs have the ability to downregulate c-Myc expression and repress the activity of glycolysis-associated genes, including GLUT1 and HK2. Consequently, GNPs effectively reduce enzymatic activities associated with glucose consumption, ATP production, lactic acid production, glucose transport (GLUT1), and glucose phosphorylation (HK2) in tumor cells. Ultimately, the deprivation of energy due to GNPs leads to the demise of tumor cells.

Tumor necrosis factor (TNF), also referred to as TNFα, is a multifaceted cytokine that holds paramount importance in cellular functions such as survival, cell division, differentiation, and apoptosis [33]. Its secretion predominantly occurs in activated macrophages and dendritic cells, and it has been implicated in inflammation-associated carcinogenesis. The inflammatory effects of TNF are predominantly mediated through its binding to TNF Receptor 1 (TNFR1) and subsequent activation of the NF-κB pathway. Perturbation of TNFR1-NF-κB signaling has been shown to neutralize the inflammatory effects of TNF and induce cell death. Studies have indicated that Gold (I) compounds possess immunosuppressive properties by inhibiting the activation of the IB kinase and inducing apoptosis [34]. Moreover, Au(I) compounds have demonstrated the ability to regulate TNF-α levels by modulating the activation of specific immune cells, including neutrophils and macrophages. These mechanisms might explain the effectiveness of gold-based formulations in reducing TNF levels comparable to those of standard drugs. In our experimental study, we observed that the medium dose of SB exhibited significant anticancer properties by effectively reducing TNF levels (Fig. 2). This suggests that SB may possess inhibitory actions on the NF-κB pathway, thereby potentially mitigating inflammation-induced carcinogenesis. Furthermore, SB may play a pivotal role in the activation of the JNK pathway, which is responsible for apoptosis in tumor masses. However, further experiments are warranted to validate these findings and elucidate the underlying mechanisms [35].

Interleukin-6 (IL-6) is a crucial cell signaling molecule that is often found dysregulated and abundantly present in the tumor microenvironment. Our investigation revealed a progressive decrease in the circulating levels of IL-6 with escalating doses of SB. Notably, both moderate and high doses demonstrated a significant reduction in IL-6, indicating a dose-dependent response (Fig. 2). These findings are supported by previous research indicating that Gold NPs can diminish IL-6 production in Jurkat (human leukemic T cell line) and U937 (human monocytic cell line) cells [36]. GNPs have been shown to exert an antiproliferative effect on U937 cells and induce JNK-mediated apoptosis. The remarkable impact of SB on IL-6 levels may be attributed to its ability to simultaneously downregulate the expression of pro-inflammatory cytokines while upregulating anti-inflammatory molecules. This dual action impedes the survival and progression of tumor cells. Notably, IL-6 is abundantly produced by malignant cells, the tumor-associated microenvironment, CD4^+^ T cells, and tumor-associated macrophages [37,38]. By targeting the malignant cells, SB effectively lowers IL-6 levels, as demonstrated in our results with increased doses of SB.

Breast cancer cells undergoing hepatic metastasis are characterized by a multitude of intricate molecular mechanisms. In the advanced stages, these metastatic carcinoma cells display elevated levels of E-selectin ligands, which confer a significantly augmented and selective adhesion to TNFα-stimulated liver endothelial E-selectin [39].Moreover, the heightened presence of interleukin-6 (IL-6) is presumed to bolster tumor invasion and the formation of metastatic lesions [40]. The transfer of cancer cells to hepatic sites imposes detrimental effects on liver tissue, compromising its physiological functions. Evaluation of liver injury in the context of breast cancer often relies on measuring the levels of aspartate transaminase (AST) and alanine aminotransferase (ALT), both commonly employed laboratory markers for assessing liver function [41,42]. Recent investigations have indicated that an elevated AST/ALT ratio serves as an unfavorable prognostic indicator in cancer patients. In murine models, EAC tumors induce alterations in liver weight and body weight, underscoring their impact on hepatic physiology [43,44]. Notably, ethoxyquin has shown promise in inhibiting the progression of murine EAC through autophagy inhibition and LDH suppression [45]. Increased EAC tumor burden is correlated with elevated secretion of liver enzymes. The inflammatory microenvironment created by EAC cells promotes angiogenesis and the release of inflammatory growth factors and cytokines, including VEGF, MTDH, TNF-α, and TGF-β, which engage specific hepatic receptors. As cancer progresses, inflammatory cell infiltration intensifies, causing further damage to hepatic cells and the subsequent release of serum AST and ALT in EAC tumor-bearing mice. In this study, the administration of SB demonstrated a significant reduction in ALT and AST levels at lower dose only (Fig. 2) [46,47].

Weight loss and anorexia (loss of appetite) are common symptoms in advanced cancer stages that exhibit varying prevalence (39 %–81.5 % for weight loss and 30 %–80 % for anorexia. Anorexia, leading to reduced energy intake, contributes to cancer-related malnutrition and cachexia, impacting quality of life (QOL) and overall symptom distress. Multiple cancer-related peripheral factors, such as pro-inflammatory cytokines, dysphagia, nutrient alterations, hypoxia, and hormonal changes, can cause anorexia [48]. However, an experimental study revealed no reduction in body weight or decreased food intake in treatment groups, although non-significant reductions in tumour volume compared to the standard control was observed [49].The distinct food consumption pattern may be attributed to the vehicle (honey), SB, or their combined effects. While traditional Ayurvedic texts note honey's appetite-promoting activity and the biochemical details for the same are need to be explored more [50].

The above findings are reflected in the histopathological details with the normal renal cell architecture restored in the higher concentration of SB. Hepatocytes were exhibiting a normal hepatocyte radiating appearance while increasing the SB concentration. This infers that SB might be able to complement the conventional therapy as Doxorubicin is a known hepatotoxic agent [51]. The higher concentration of SB was also found to be protective towards splenic cells in the present experiment. The Histopathological images are attached in the supplementary file in the series of Fig. 5.A, B, C.

Although, the study was planned to gather some preliminary supporting evidences present in the blood of the mice model, there are still space for extensive research works. Among the lacuna, firstly, detailed histopathological changes over different doses are not studied. Most importantly, molecular basis of cellular intrinsic mechanism of SB are not studied that could provide concrete evidences of its anti-tumour capabilities. However, the probable Pharmacokinetic and Pharmacodynamics mechanism of SB has been represented as a flow chart in Fig. 6 in the Supplementary File.

## 4 Conclusion

*Swarna Bhasma,* containing gold demonstrates a dose dependent capacity to combat cancer. Here a concentration dependent effectiveness might have been linked to the specific pathways involved to redress the tumor volume. Specifically in this experiment, SB demonstrated the ability to decrease the blood concentration of tumor specific markers such as CEA, TNF-alpha, IL-6 and liver function markers (including ALT and AST) in the treated groups. The potential anti-tumor effects of SB could be attributed to the following mechanisms (1) Suppression of CEA levels within the tumor mass, indicating that SB might hinder the expression of c-Myc and induce an energy-deprived state by downregulating genes associated with glycolysis, such as GLUT1 and HK2, (2) Effective reduction in TNF levels suggests that it might counteract inflammation-mediated carcinogenic changes by interfering with the NF-κB pathway, as well as promote apoptosis by activating the JNK pathway, (3) Notably, a dose-dependent decrease in IL-6 levels was observed at both moderate and high doses of SB. This reduction may be attributed to the simultaneous downregulation of pro-inflammatory cytokines and the upregulation of anti-inflammatory molecules, contributing to its potential in mitigating inflammation-associated with cancer, (4) SB significantly decreases liver parameters and alters the AST/ALT ratio positively in lower doses, whereas higher doses are with elevated AST/ALT ratio. Although the provided evidence sheds light on the anti-carcinogenic activities of SB, further comprehensive investigation focusing on pathway-based evidence is crucial to gain a deeper understanding and validate its remarkable potential in combating cancer.

## Author contributions

Conceptualization, Methodology and Study Design-NJ; Application of software-PK, SS, MKD; Validations of the data- NJ, PK, SS, MKD; Data curation, Reviewing, drafting and editing -NJ, PK, SS, MKD, RJ; Histological Evaluation-AM, HV, RJ.

## Declaration of generative AI in scientific writing

Authors have not used any AI tools in the scientific writing of the manuscript.

## Funding sources

None.

## Declaration of competing interest

The authors declare that they have no known competing financial interests or personal relationships that could have appeared to influence the work reported in this paper.
