# Supplementary material for: Swarna Bhasma reduces the blood concentration of tumor-specific signatures and protects from hepatocellular damages in Ehrlich ascites mice model
Source: J Ayurveda Integr Med. 2025 Jul 24;16(5):101152. doi: 10.1016/j.jaim.2025.101152 (PMC12309017; doi:10.1016/j.jaim.2025.101152)
Supplement: Multimedia component 1 [file mmc1.docx]

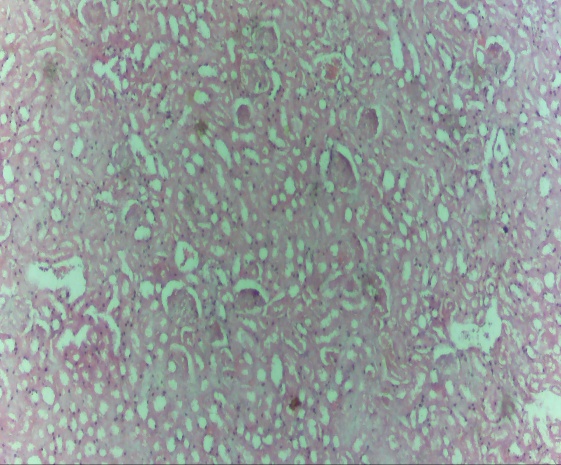

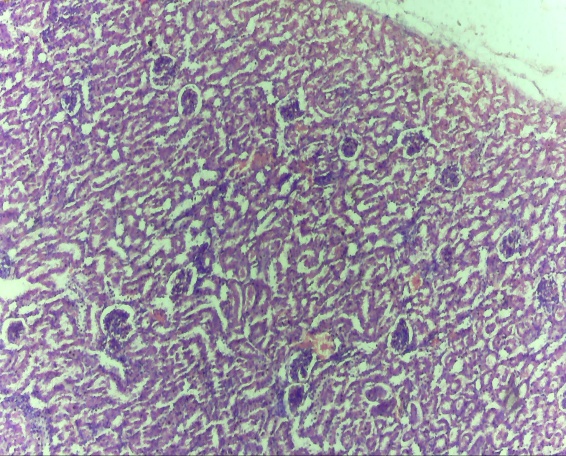


**Fig.5.A.** Images of H&E stained Renal structure of positive control and SB treated groups

Group 3

Group 2


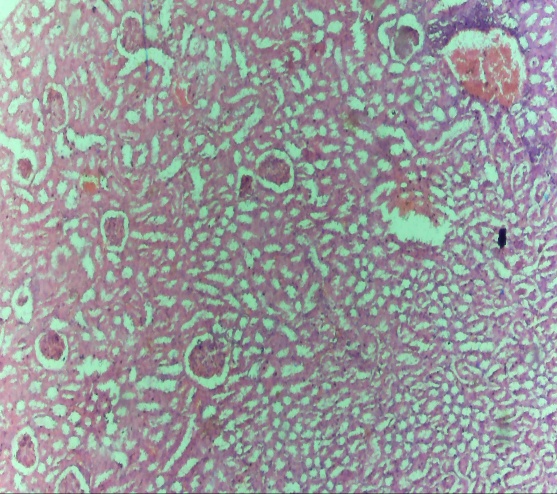

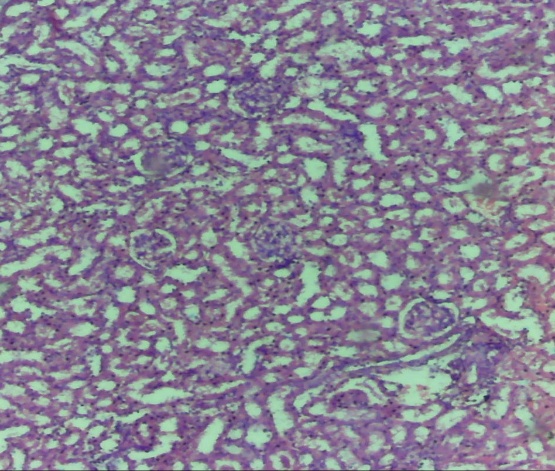


Group 4

Group 5

(H & E) 4 X magnified images of Group 2, 3, 4, 5, & 6

**Group 2**: Shrunken glomerulus and coalesced multinucleated Proximal Convoluted Tubules (PCT)

**Group 3**: The cyto-architecture of normal renal form is lost and the arrow indicates the preserved glomerulus.

**Group 4**: Medullary region with polymorph nucleated PCT.

**Group 5**: Abnormal cyto-architecture with coalesced glomerulus.

**Group 6**: The cyto-architecture is restored with normal PCT and Distal Tubule (DT) lining.


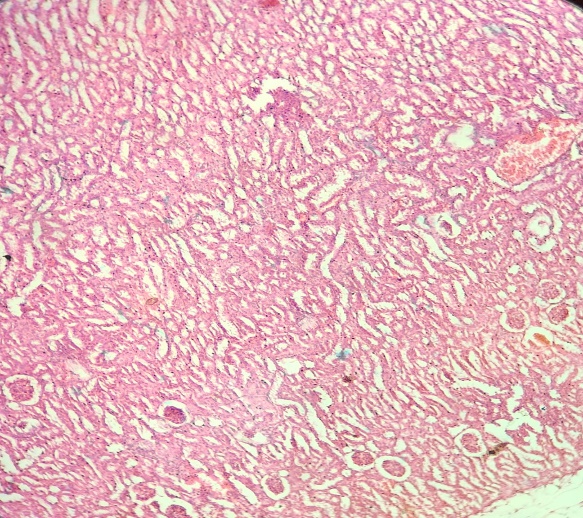


Group 6

**Fig.5.B** Images of H&E stained Hepatic cells of positive control and SB treated groups


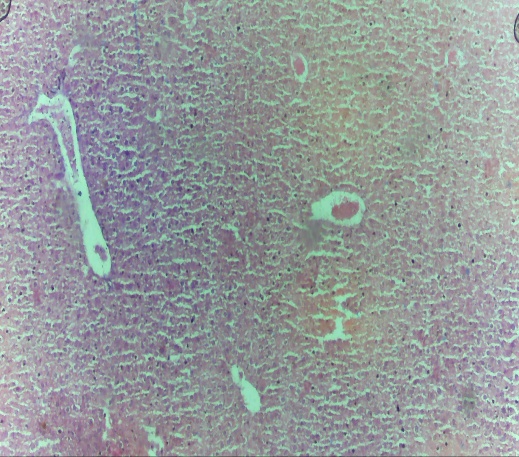

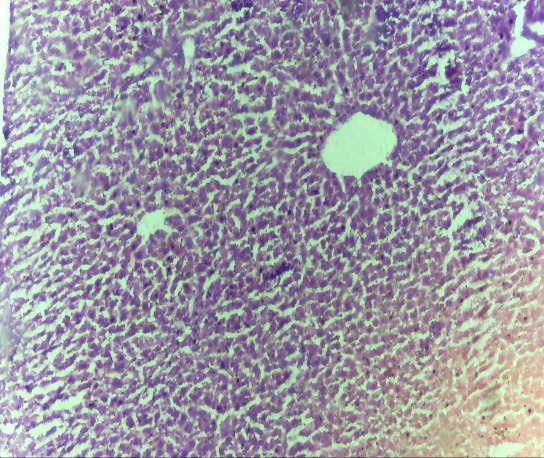


Group 3

Group 2


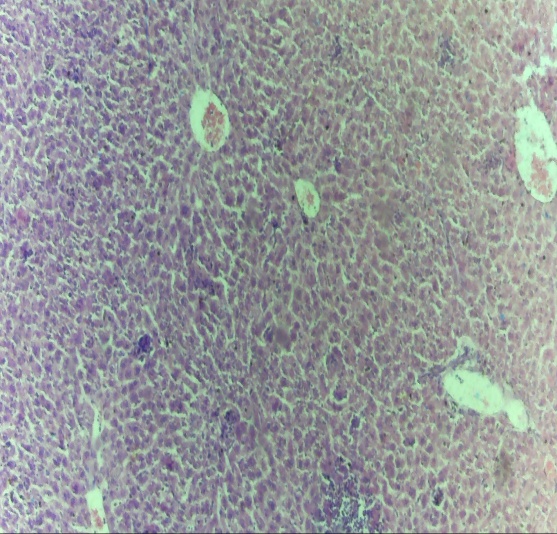

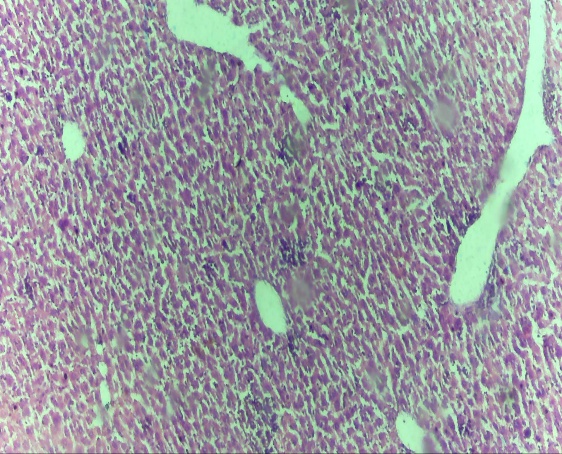


Group 5

Group 4

H & E 4X magnified images of Group 2, 3,4,5 & 6.

**Group 2**: Derangement of radiating hepatocytes due to disease pathogenesis.

**Group 3**: The hepatocytes are seen in coalesced form.

**Group 4**: Central vein with hemorrhage and restoration of hepatocyte radiating appearance.

**Group 5**: Arrow indicates the glycogen deposition and the vacuolations created from it.

**Group 6**: The cyto-architecture is seen as restored with normal radiating hepatocytes from the central vein.


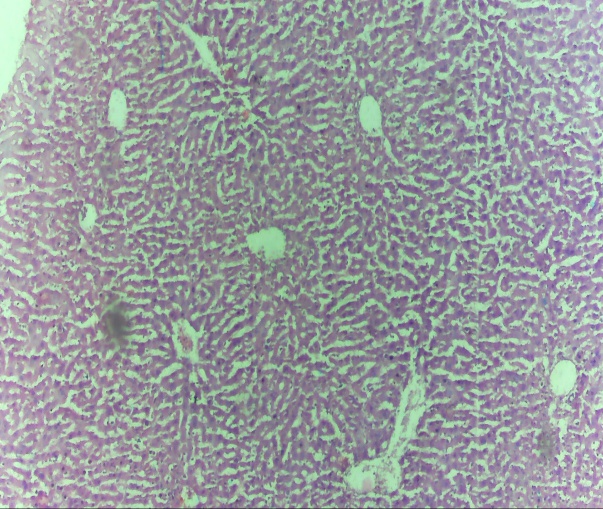


Group 6

**Fig.5.A.**C.Images of H&E stained Splenic cells of positive control and SB treated groups


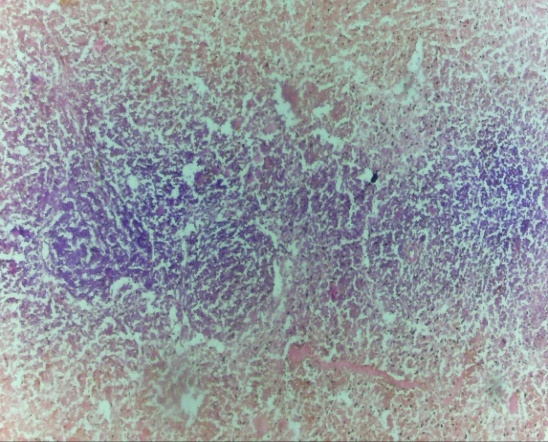

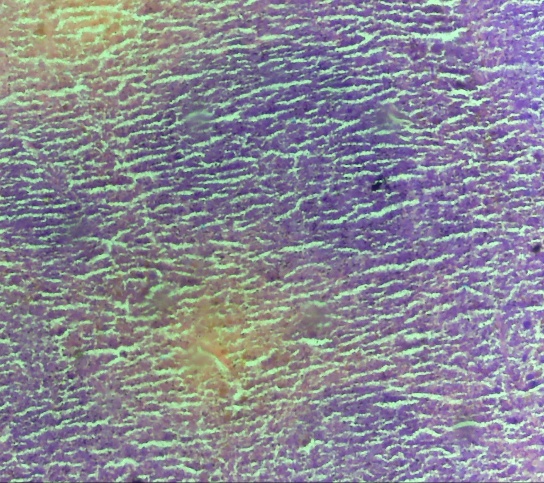


Group 3

Group 2


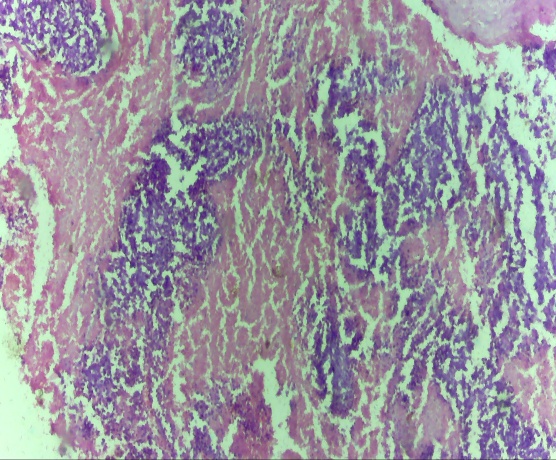

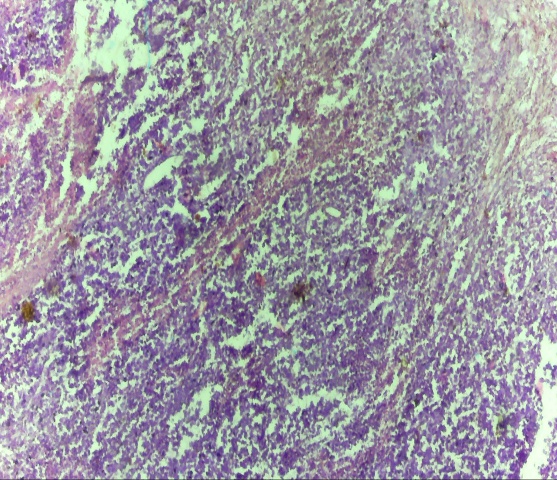


Group 4

Group 5

H & E 4 X magnified images of Group 2, 3, 4, 5 & 6

**Group 2**: arrow mark points the poly-morpho nucleated coalesced splenic cells.

**Group 3**: A distinguished red and white pulp areas with abnormal cellular architecture.

**Group 4**: Arrow indicates the abnormal presentation of splenic cells.

**Group 5**: Preserved red and white pulp regions with septal deviations.

**Group 6**: Well demarcated red and white pulp regions exhibited.


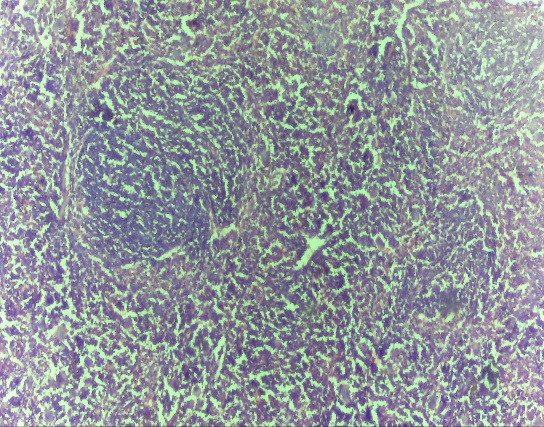


Group 6

**Table no: 4 Tumour volume of the all the groups in the 0,7^th^ ,14^th^ and 21^st^ day**

|  | **0 day** | **7^th^ day** | **14^th^ day** | **21^st^ day** |
| --- | --- | --- | --- | --- |
| **VC** | 512.42 ± 236.39 | 570.89. ± 288.64 | 630.43. ± 271.28 | 680.57± 285.92 |
| **DC** | 382.05. ± 103.88 | 423.78. ± 91.37 | 546.46. ± 86.03 | 662.64 ±145.66 |
| **SC** | 259.43. ± 108.65 | 258.69. ± 106.00 | 236.34. ± 105.35 | 204.55± 90.51 |
| **TL** | 488.83±183.25 | 518.08±185.01 | 511.60±197.23 | 492.03±194.76 |
| **TM** | 337.38 ± 149.64 | 289.95± 156.61 | 182.33±68.02*** | 83.70±48.14*** |
| **TH** | 410.19±118.68 | 199.28±58.02 | 81.25±28.24**** | 20.69±11.07**** |

n=4 and p <.05*, p<0.01**, p<0.0001*** and p<0.0001**** are considered significant. VC-Vehicle control, DC-Disease control, SC-Standard control, TL-Tumour volume in lower dose, TM- Tumour volume in middle dose and TH-Tumour volume in higher dose.

**Figure 6:- Flow chart representing the Probable Pharmacokinetic and Pharmacodynamics of SB**

**Pharmacokinetics of *Swarna Bhasma***

*Swarna bhasma*

Absorption

Sublingual and gastro intestinal, Patil-Bhole,T et al,2018

 Below 20nm via clathrin-dependent receptor-mediated endocytosis (AuNPs) and above 100nm with macropinocuytosis.( human foreskin fibroblast model) (Beaudet D et al, 2017)

Blood compatibility aids the minerals and phytochemicals to be target specifically delivered (Sonavane,,et al, 2008).

Distribution

Bioavailability depending on the particle size above 100nm due to the loading effect Patil-Bhole T et al 2018

Metabolism

The larger Nano particles (60 to 100nm) are safely excreted via kidney and from liver via bile and faeces. Less than 20 nm might be accumulating in the brain and lungs.(WIli Paul et al, 2011)

Excretion

**Pharmacodynamics of *Swarna Bhasma***

SB might be aiding the neuronal stimulation with Ca^2+^influx and supporting the conductivity at synaptic junctions (Paviolo et al, 2013)

SB can scale up the SOD (Superoxide dismutase) and Catalase enzymes to aid the free radical piling up. (A.Mitra et al 2003)

*Swarna bhasma* at the target site

In tumors, decreased the CEA, (Mueller, R et al 2020), IL-6 (John Benjamin.W et al 2023), the GLUT-1, (Li, J.et al 2024) ATP production (Chen, YC. Et al 2025) and TNF- α (Visaria, R. K. et al) levels.
